# Glioblastoma invasion and cooption depend on IRE1α endoribonuclease activity

**DOI:** 10.18632/oncotarget.4679

**Published:** 2015-07-23

**Authors:** Arnaud Jabouille, Maylis Delugin, Raphaël Pineau, Alexandre Dubrac, Fabienne Soulet, Stéphanie Lhomond, Nestor Pallares-Lupon, Hervé Prats, Andreas Bikfalvi, Eric Chevet, Christian Touriol, Michel Moenner

**Affiliations:** ^1^ Inserm, U1029, 33400 Talence, France; ^2^ Univ. Bordeaux, 33000 Bordeaux, France; ^3^ Inserm, U1037, CHU de Rangueil, 31432 Toulouse, France; ^4^ Inserm, U1053, 33000 Bordeaux, France; ^5^ Centre Régional de Lutte Contre le Cancer Eugène Marquis, 35000 Rennes, France; ^6^ ER440, « Oncogenesis, stress, signaling » Univ. Rennes 1, Rennes, France; ^7^ CNRS UMR5095, IBGC, 33700 Bordeaux, France

**Keywords:** Pathology Section, glioblastoma, angiogenesis, invasion, perivascular growth, mesenchymal differentiation

## Abstract

IRE1α is an endoplasmic reticulum (ER)-resident transmembrane signaling protein and a cellular stress sensor. The protein harbors a cytosolic dual kinase/endoribonuclease activity required for adaptive responses to micro-environmental changes. In an orthotopic xenograft model of human glioma, invalidation of IRE1α RNase or/and kinase activities generated tumors with remarkably distinct phenotypes. Contrasting with the extensive angiogenesis observed in tumors derived from control cells, the double kinase/RNase invalidation reprogrammed mesenchymal differentiation of cancer cells and produced avascular and infiltrative glioblastomas with blood vessel co-option. In comparison, selective invalidation of IRE1α RNase did not compromise tumor angiogenesis but still elicited invasive features and vessel co-option. *In vitro*, IRE1α RNase deficient cells were also endowed with a higher ability to migrate. Constitutive activation of both enzymes led to wild-type-like lesions. The presence of IRE1α, but not its RNase activity, is therefore required for glioblastoma neovascularization, whereas invasion results only from RNase inhibition. In this model, two key mechanisms of tumor progression and cancer cell survival are functionally linked to IRE1α.

## INTRODUCTION

Inositol-requiring enzyme 1α (IRE1) is an endoplasmic reticulum (ER)-resident type I transmembrane protein and a sensor of the Unfolded Protein Response (UPR). The protein harbors both intrinsic Ser/Thr kinase and endoribonuclease (RNase) activities in its cytoplasmic domain. IRE1α is ubiquitously expressed in mammalian tissues and is an essential stress sensor at the convergence point of numerous signal inputs handled by cells. It coordinately regulates anabolic and catabolic processes and also conveys survival or death signals depending on the magnitude or duration of the ER stress [1–4].

The characteristics and domain organization of IRE1 are essentially conserved in eukaryotes [5–7]. Upon accumulation of misfolded proteins in the lumen of the ER, IRE1α proteins undergo a transition from inactive monomeric forms to active oligomers [2, 6–8], leading to trans-autophosphorylation and RNase activation [2, 3, 6, 7]. Mammalian IRE1α RNase domain cleaves a variety of RNAs, a process named “regulated IRE1-dependent decay” (RIDD) [9, 10], and also initiates by a different mechanism the selective and unconventional cytoplasmic splicing of the X-box binding protein-1 (XBP1) mRNA [11]. The spliced XBP1 mRNA is translated into a transcription factor involved in the modulation of the UPR and adaptive response to stress, including ischemia [11–13]. A second signal transduction cascade mediated by IRE1α relies upon the recruitment of the multifunctional adaptor protein TRAF2, leading to the activation of JNK and NF-κB transduction pathways [1, 14, 15].

IRE1α was shown to contribute to tumor development through its RNase activity [13, 16–19]. Non-random somatic mutations of IRE1α were also reported in a variety of neoplastic tissues [20]. Moreover, inactivation of IRE1α by using dominant-negative approaches or siRNA-mediated knockdown led to the decrease of pathological angiogenesis in a human glioblastoma xenograft model and also drove mesenchymal differentiation and invasive mechanisms [21]. The latter observation is of particular interest, as the mesenchymal profile characterizes a major subclass of high-grade gliomas frequently observed upon recurrence [22, 23].

Interestingly, angiogenesis and invasiveness are functionally linked and, to a certain extent, mutually exclusive [24]. Most importantly, inhibition of angiogenesis may favor malignant cell dissemination into the surrounding normal tissues, which represents an insidious and devastating feature of glioma development [24–26]. It is therefore crucial to clarify the nature of the molecular inter-relationships linking these two processes in order to optimize antitumor therapy.

Here, we examined in more details the contribution of IRE1α kinase and RNase activities in glioblastoma angiogenesis and invasion. Inducible and selective invalidation of either or both catalytic activity was achieved in malignant cells, and the resulting effects were analyzed in an orthotopic mouse xenograft model. Several key parameters of high-grade glioma progression were monitored, such as the recruitment of new blood vessels, infiltration of cancer cells and apoptosis. We show that the selective inhibition of IRE1α RNase activity favors glioblastoma invasion and blood vessel co-option. In addition, we establish a correlation between the presence of a functional IRE1α kinase domain and the occurrence of tumor angiogenesis.

## RESULTS

### Production and characterization of IRE1α catalytic mutants

**Figure 1:**
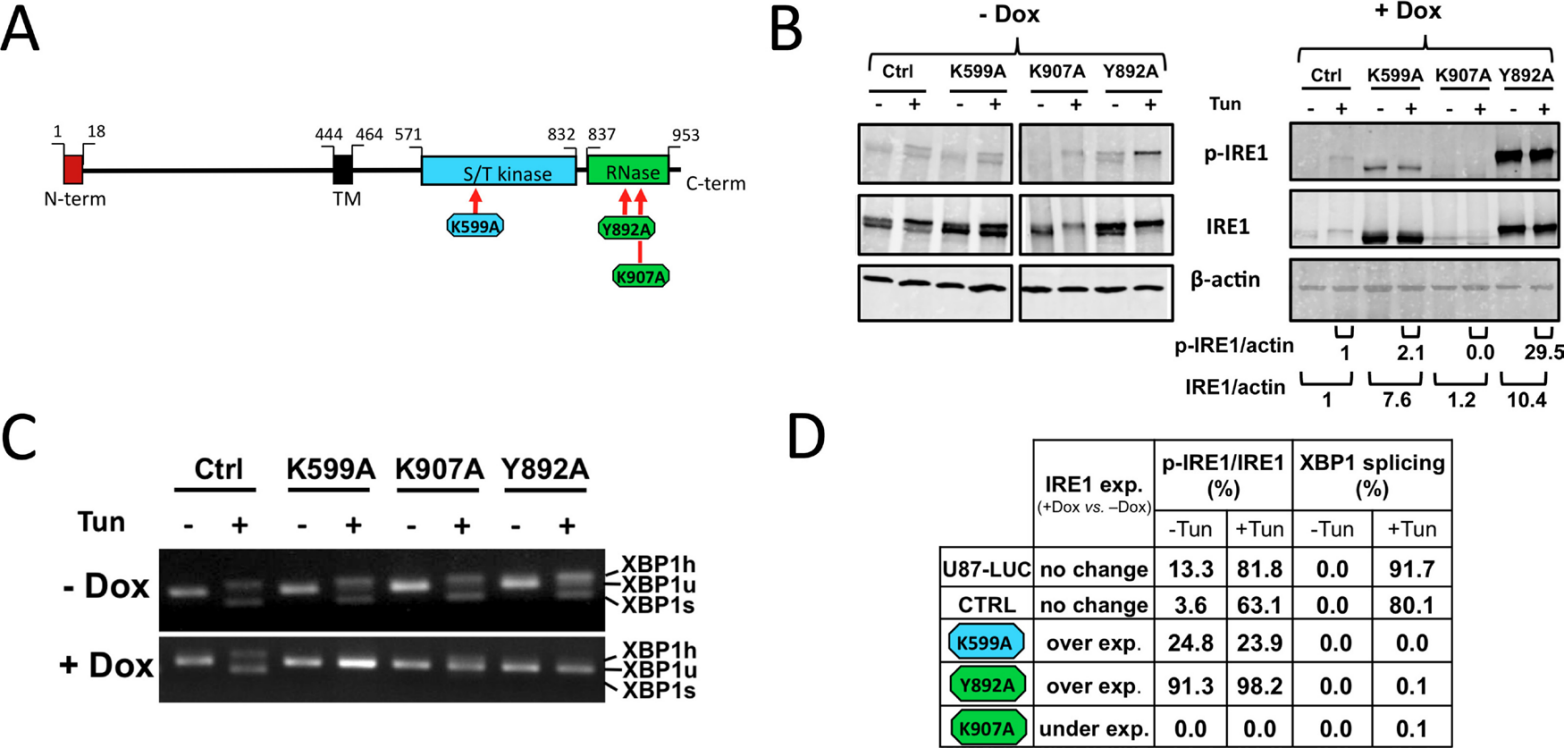
Alteration of the enzymatic activities of human IRE1α by site-directed mutagenesis IRE1α punctual mutants (K599A, K907A and Y892A) were expressed in U87-LUC cells under the dependence of doxycycline. **A.** Domain organization of hIRE1α protein. Point mutations were designed either at the ATP-binding site within the kinase domain (K599A) or in the IRE1α RNase domain (K907A and Y892A). **B.** Measure of IRE1α autophosphorylation. Cells were incubated for 48 h with (+Dox) or without (−Dox) 2 μg/ml doxycycline and were then stimulated or not with the ER stress inducer tunicamycin (Tun) for 2 h. Whole cell lysates were subjected to immunoprecipitation using anti-IRE1 protein antibodies. IRE1α was revealed by immunoblotting using either antibodies against phospho-(Ser724)-IRE1α (p-IRE1) or against total IRE1α (IRE1). β-actin was used as internal control. Pixel intensities of IRE1α proteins normalized to actin, and ratios of phosphorylated Ser-724 IRE1α (p-IRE1) to total IRE1α are indicated. **C.** Inhibition of XBP1 splicing. Cells grown with or without doxycycline were stimulated or not with tunicamycin. XBP1 transcripts were detected by PCR after reverse trancription using primers flanking the mRNA splicing sites: XBP1u, unspliced mRNA doublet; XBP1s, spliced doublet; XBP1 h, unspliced/spliced hybrid. **D.** IRE1 expression and relative kinase and RNase activities. IRE1 kinase activity was reported in percent of p-IRE1α to total IRE1α. RNase activity was given in percent of total XBP1 splicing.

Three catalytic mutants of IRE1α were generated by site-directed mutagenesis, including a kinase-inactive variant (K599A) and two endoribonuclease-inactive variants (Y892A and K907A) (Figure 1A). U87 glioblastoma cells were transduced with an inducible Tet-ON vector expressing the different IRE1 mutants or GFP under the control of doxycycline (Figures 1 and S1A–S1AB). Wild-type IRE1α protein was also expressed in control cells (Figure S2A). Sustained expression of the three mutants did not impede tumor cell viability and neither PERK/eIF2α nor ATF6 branches of the UPR were activated in these conditions. Indeed, neither eIF2α nor phospho-eIF2α protein levels were significantly altered following incubation with doxycycline (Figure S1D). Similarly, ectopic expression of the different IRE1α protein mutants did not induce the translocation of a FLAG-tagged ATF6α protein from the ER membrane into the nucleus, as observed upon stress condition (Figure S3).

Under doxycycline treatment, expression of IRE1α-K599A and IRE1α-Y892A protein variants were readily detected, whereas that of IRE1α-K907A protein was not (Figure 1B). In addition, IRE1α phosphorylation at Ser724 (p-IRE1), a marker of protein trans-autophosphorylation, was observed in cells expressing the Y892A RNase mutant and was also present at a much lower level in K599A mutant cells (Figures 1B and 1D). In comparison, IRE1α kinase activity was not detectable in cells expressing the IRE1α-K907A mutant (Figures 1B, 1D), even though both endogenous and ectopic IRE1α transcripts were present in these cells (Figure S1B). IRE1α RNase activity was monitored using the splicing of pre-XBP1 mRNA as readout (Figure 1C). As expected, substitutions of amino-acids residues at positions 599, 892 and 907 by alanine resulted in a substantial inhibition of XBP1 splicing (>90%) (Figures 1C and 1D). Thus, expression of each of the three variants strongly impaired IRE1α RNase activity toward the splicing of XBP1 mRNA.

### Expression of IRE1α kinase and RNase variants in tumor cells reduces tumor growth and improves mouse survival

**Figure 2:**
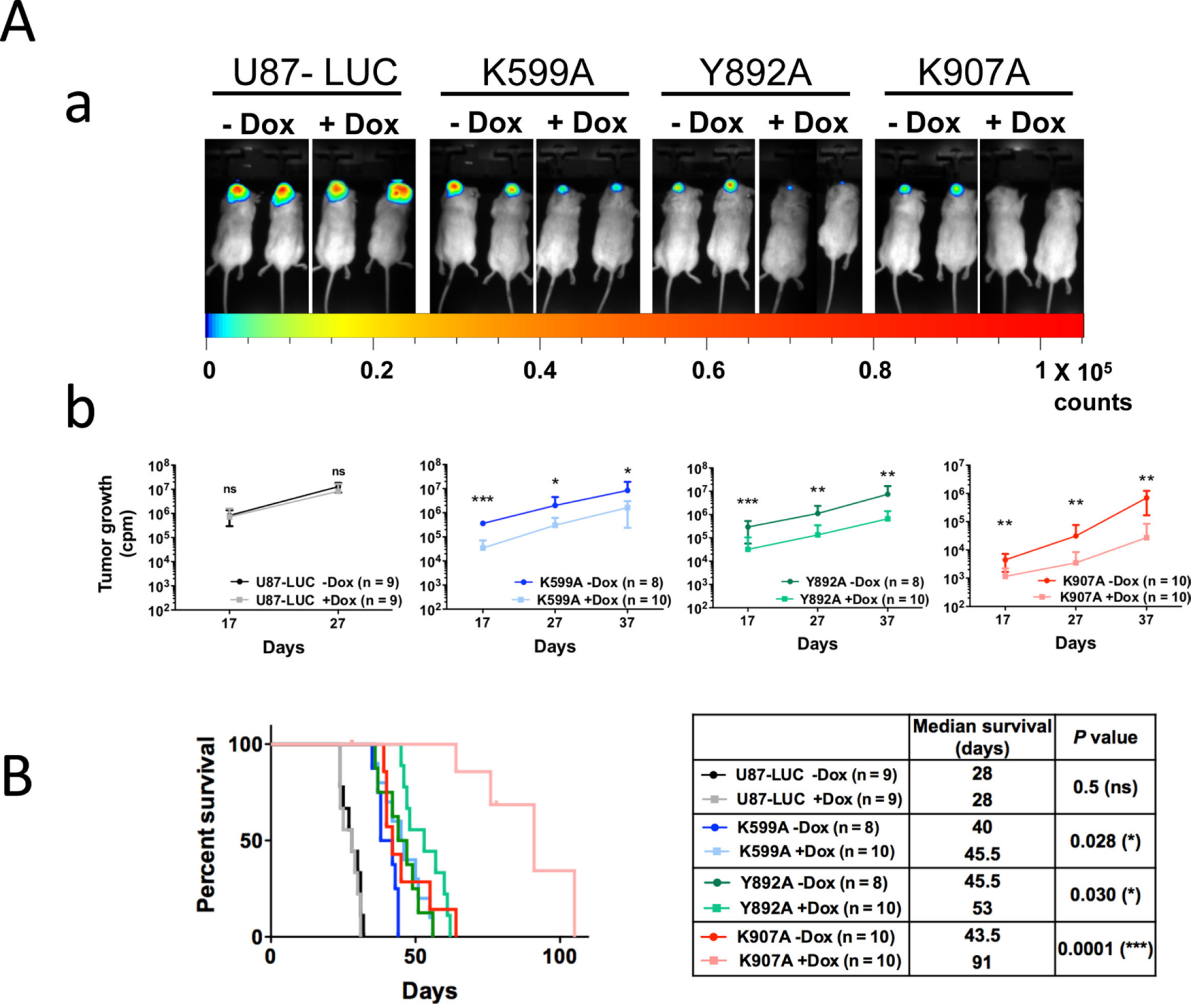
Inactivation of IRE1α kinase or IRE1α RNase activities impedes glioma growth IRE1α cell mutants were stereotactically implanted into mouse brains and tumor progression was measured at different time-intervals by imaging bioluminescence in living mice fed or not with doxycycline. **A.** Luciferase real-time imaging of U87-LUC, U87-K599A, U87-Y892A and U87- K907A-tumors. Luciferase activity was quantified after selection of the region of interest and tumor volumes were measured at days 17, 27 and 37. **Aa.** Bioluminescence imaging at day 27. **Ab.** Time-course evaluation of tumor growth for each cohort. Values are the mean ± SD. Three independent experiments were performed with similar results. **B.** Kaplan-Meier survival analysis after intracranial implantation of U87-LUC, U87-K599A, U87-Y892A or U87-K907A cells. Left panel, Kaplan-Meier plot. Right panel, characteristic values for each glioma subtype. (n, number of mice; *P < 0.05; ***P < 0.001; ns, *P* ≥ 0.05).

U87-K599A, U87-Y892A, U87-K907A and U87-LUC cells were implanted in the brain of RAG2/γ_c_ mice. Doxycycline was delivered *in vivo* in drinking water for half of the animals and glioblastoma progression was monitored at days 17, 27 and 37 by measure of the intra-tumoral luciferase activity (Figure 2). No difference in malignant growth was observed with U87-LUC control cells in the presence or absence of doxycycline (Figure 2A). In comparison, glioblastomas expressing either the K599A or Y892A variants exhibited a ≈10-fold volume reduction under doxycycline treatment from day 17 to day 37, and a ≈100-fold volume reduction was obtained with the K907A mutant at day 37. Congruent with growth measures, median survival of mice engrafted with cells expressing IRE1α-K599A, IRE1α-Y892A or IRE1α-K907A mutations increased respectively by 5 (12%), 7 (14%) and 48 (109%) days as compared to their cognate controls without doxycycline (Figure 2B).

**Figure 3:**
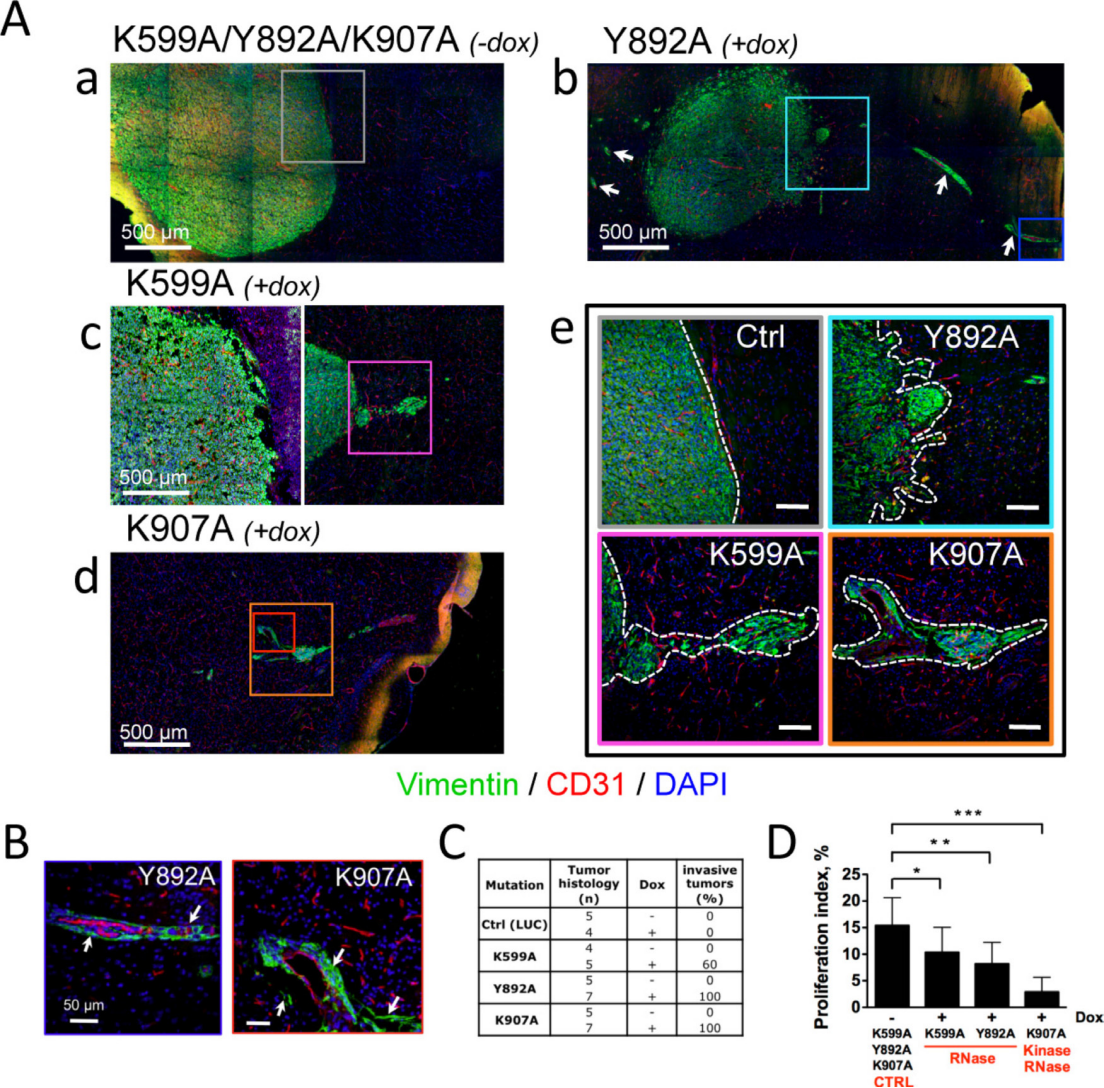
Glioblastoma neovascularization and invasion depend on IRE1α catalytic activities Glioblastoma cells bearing IRE1α mutants were xenografted into mouse brains and animals were fed with (+dox) or without (−dox) doxycycline. **A.** Invasive behavior of U87-K599A, U87-Y892A and U87-K907A-cell-derived tumors under doxycycline treatment. **Aa-e.** Coronal sections of mouse brains at day 28 (a, b, c) and day 47 (d) of tumor development were co-labeled using anti-vimentin (malignant cells, green labeling) and anti-CD31 (blood vessels, red labeling) antibodies. DAPI-labeled nuclei are in blue. Aa) Control tumors were massive and angiogenic. Ab) U87-Y892A cells developed into angiogenic and highly invasive gliomas. Perivascular glioma cell clusters (white arrows) were observed far away from the tumor core. Ac-d) Infiltrative phenotypes observed with U87-K599A cells and K907A cells. Ae) Higher magnification of boxed-areas in Aa-d. Dashed lines show borders between tumor tissues and normal brain tissues. (Scale bars: 100 μm.) **B.** U87-Y892A and U87-K907A glioma cells co-opted blood vessels. Higher magnifications of blue and red boxed-areas in Ab and Ad, respectively. **C.** Histological scoring of invasive tumors in each cohort. Any tumor responding to one of the three following criterias was scored as invasive: *i)* presence of a loosely delineated glioma rim, *ii)* existence of micro-satellites (*n* ≥ 10) in the periphery of the bulky core, and *iii)* emergence from the tumor core of extensions whose lengths were equal or superior to 500 μm. **D.** Measure of the proliferation index by using Ki-67 labeling. The percentage of dividing cells was obtained from the counting of at least 5, 000 nucleis for each tumor group (see also Figure S4A).

Mutations invalidating either the IRE1α kinase or RNase therefore led to decreased glioblastoma growth and to improved outcome, the best effect being obtained with the K907A mutant. Higher median survival times correlated well with the decrease in tumor cell proliferation (Figures 3D and S4A), U87 parental tumors showing a greater mitotic index (∼15%) than cells expressing IRE1α mutant cells (10.0%, 7.7% and 2.5% of Ki-67 positive cells for K599A, Y892A and K907A variants, respectively). Apoptosis was not involved in the reduction of glioblastoma growth as shown by real-time imaging (Figure S4B) and by counting pyknotic nuclei in tumor sections (*n* < 5% of total neoplasic cells in either conditions).

### Selective inhibition of IRE1α RNase activity promotes glioblastoma invasiveness without affecting microvascular proliferation

The morphology of tumors formed by cells expressing IRE1α variants was examined by immunofluorescence microscopy at day 28 (K599 and Y892A mutants) or at day 42 (K907A mutant) post-implantation. In the absence of doxycycline (Figures 3Aa and S5), all tumors exhibited typical characteristics of compact, homogenous, well-delineated and aberrantly vascularized tissues. The phenotype was also observed in tumors overexpressing an ectopic IRE1wt protien (Figure S2). Under doxycycline treatment, the IRE1α-Y892A mutation generated bulky, highly vascularized and loosely cohesive lesions (Figures 3Ab and 3Ae). These tumors were also diffuse, poorly delineated, and elaborated an invasive network by colonizing perivascular areas of normal blood vessels (white arrows) from the immediate vicinity of the tumor core up to distal sites (Figures 3Ab and 3B). In comparison, U87-K599A-derived glioblastomas exhibited intermediate features of invasion, with a lower penetrance (∼60% of the tumors presented infiltrative contours; Figure 3Ac and 3C) and no detectable vessel co-option. Finally, U87-K907A-derived glioblastomas that did not significantly express IRE1α were essentially avascular, developed at a much slower rate, and presented typical features of invasion with perivascular cuffing (Figures 3Ad, 3Ae, 3B and 3C). The re-arrangement of the tumor shape observed in U87-Y892A- and U87-K907A-derived tumors confirmed our previous observations in glioblastomas deficient for IRE1α signaling [21].

**Figure 4:**
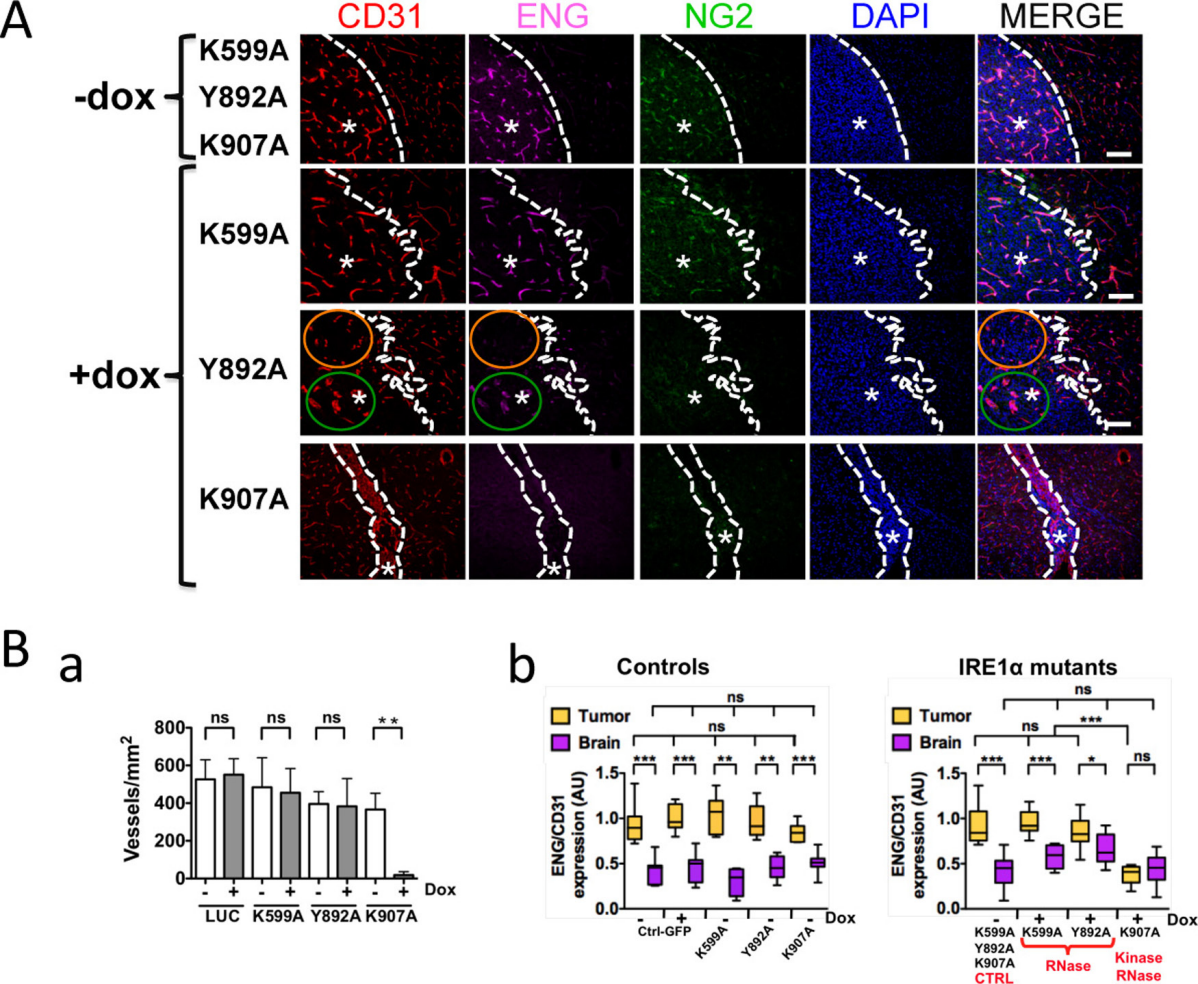
IRE1α RNase deficiency promotes growth of angiogenic and invasive Glioblastomas Glioblastoma cells were xenografted into mouse brains. IHC labeling was then performed on brain coronal sections using antibodies directed against CD31 (total blood vessels), ENG (endoglin; angiogenic vessels) and NG2 (pericyte-like cells). **A.** Representative labeling of CD31, ENG and NG2 antigens. DAPI-labeled nuclei are in blue. Dashed lines delimit the border between normal and neoplastic (asterisks) tissues. Intra-tumoral tissue heterogeneity of IRE1 Y892A tumors was pointed out in two distinct areas: the orange circle delimits a co-opted vessels area as depicted by positive CD31 staining and negative ENG staining; the green line encircle an extensively angiogenic area positive for both CD31 and ENG stainings. **B.** Quantification of the neovascularization pattern in glioma tissues and in the proximal brain parenchyma. **Ba.** The total number of blood vessels was quantified by using CD31 labeling. Four different sections were analyzed per tumor and three to eight tumor samples were analyzed for each mutant. **Bb.** Proliferative blood vessels were represented by measure of the two-protein ratios (ENG *vs*. CD31) and signal intensities were expressed in arbitrary units (AU) as box and whiskers plots. Left, control conditions; Right, effect of IRE1α mutations (−Dox, +Dox). Control values (−Dox) were presented as pooled data from K599A, Y892A and K907A tumors. Fluorescence signals were quantified from four different tumors and at least two independent implantations. Comparisons between paired-samples were analyzed by using the Mann–Whitney test. Statistical analyzes including several unpaired samples were performed by using the Kruskal–Wallis test (*P < 0.01; **P < 0.005; ***P < 0.001; ns, not significant).

Triple immunofluorescence staining was then performed to better characterize the vascular pattern of these tumors (Figure 4A). Proliferative blood vessels and vessel density were quantified by labeling endoglin (ENG) and CD31, respectively, and their expression ratios (ENG *vs*. CD31) in tumors were compared to values obtained from the cerebral vascular bed (Figures 4B and S6). These markers were expressed at a low level in the quiescent brain vasculature. In comparison, control neoplasms as well as U87-K599A- and U87-Y892A-derived tumors were highly angiogenic, as depicted by positive CD31 and ENG staining. A robust NG2 proteoglycan immunoreactivity, a consistent marker of high-grade gliomas [27], was also observed. Localized angiogenic “hot spots” were detected within U87-Y892A-derived tumors (Figure 4A, green circle) and were sometimes regionally distinguished at a distance of only a few micrometers from vessel-co-opted areas (orange circle). Comparatively, U87-K907A-derived tumors were essentially avascular (Figure 4Bab). Thus, inhibition of the neovascularization process was only detected in IRE1α mutants deficient for both kinase and RNase activities (Figures 4A–4B). Notably, the non-angiogenic and co-opted phenotype of U87-K907A-derived tumors was maintained over time (up to 78 days post-implantation; Figure S6). Hence, no redundant angiogenic signaling emerged from invalidation of both catalytic domains of IRE1α in this time interval.

### IRE1α RNase deficient glioblastomas exhibit mesenchymal features

**Figure 5:**
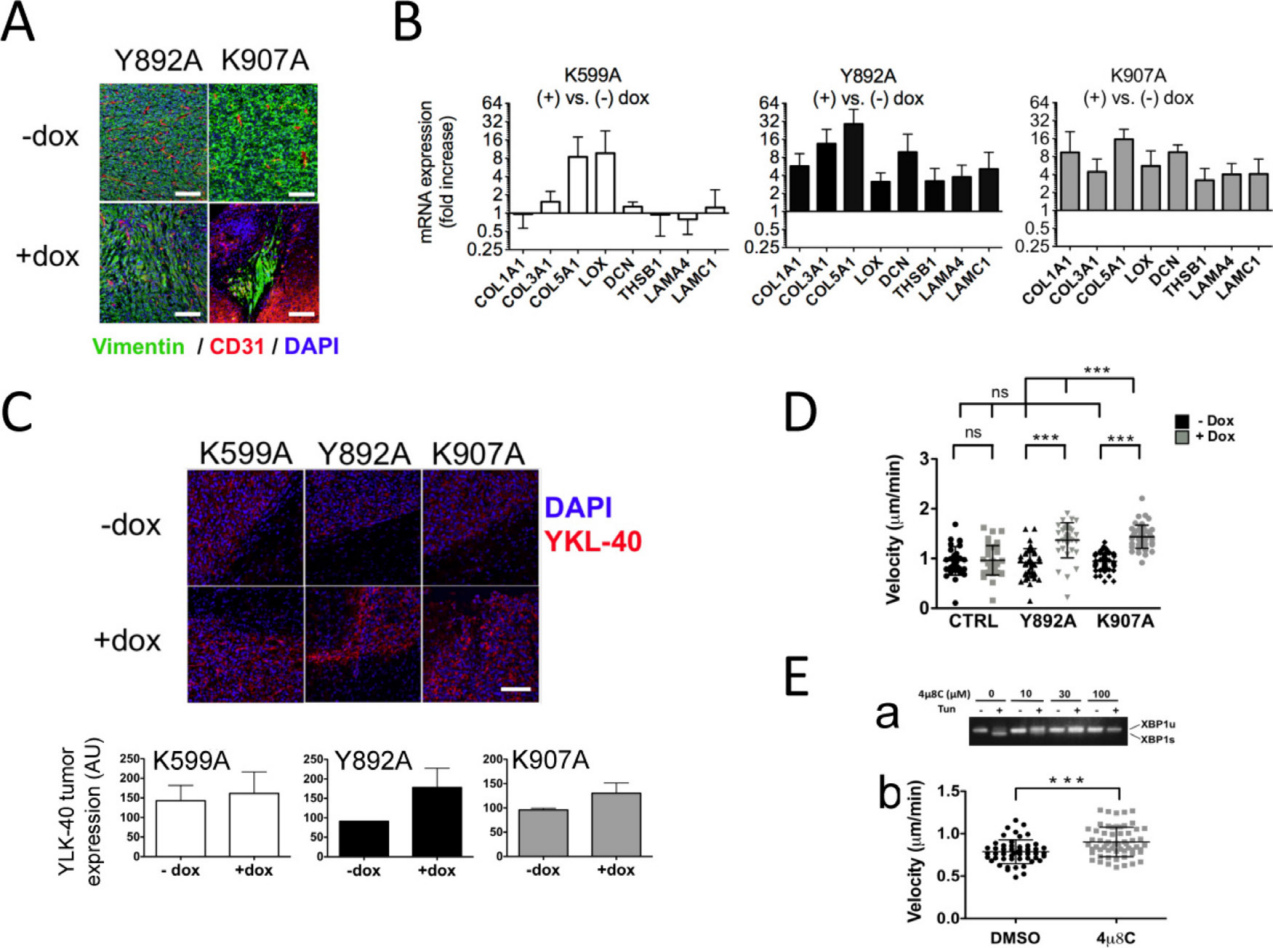
IRE1α RNase inhibition induces mesenchymal differentiation of glioblastoma cells and increases chemotaxis **A.** U87-Y892A and U87-K907A cells adopted a mesenchymal phenotype. U87-Y892A and U87-K907A-derived tumors were grown intracranially for 28 and 47 days, respectively. **B.** Mesenchyme-specific genes were expressed in IRE1α RNase-defective gliomas. Brain tissues (*n* ≥ 5 for each condition) were analyzed individually after 4 weeks of tumor development. RTqPCR analysis was carried out on a series of representative genes of the cellular matrix and of mesenchymal differentiation. Expression of the transcripts was given as fold increases in gliomas treated *versus* untreated with doxycycline (mean value ± SD). HPRT1 and β-actin were used as reference genes. **C.** Detection of YKL-40 antigen. Fluorescence intensity was quantified on two different fields of each tumor (center and rim). Three independent samples were analyzed in each group. (Bar = 100 μM.) **D.** Chemotaxis assays using live-cell imaging. Cells were seeded in the observation area of chemotaxis plates. One chamber was filled with DMEM/FCS/glucose, and the other with DMEM only. Individual cells were visualized in each field and chemotaxis was monitored by time-lapse for 12 h with photos taken every 15 min. Results are expressed as the average velocity (μm/sec). **Ea.** 4 μ8C inhibits IRE1α nuclease activity in wild-type U87 cells. Cells were exposed for 24 h to increasing amounts of 4 μ8C and presence of XBP1u and XBP1s transcripts was analyzed. **Eb.** 4 μ8C increased cell motility. Cells were seeded in the observation area and the two opposite chambers were filled respectively with DMEM/FBS/glucose and DMEM only. The 4 μ8C compound (20 μM) was added in both chambers. A control assay was performed in the presence of the DMSO solvent. Statistical analyzes were performed by using the Mann–Whitney test (***P < 0.001; ns, not significant).

The diffuse phenotype of IRE1α-deficient glioblastomas was prominent in U87 cells expressing Y892A and K907A variants. Infiltrative cells were elongated and tumor tissues showed a fibrous aspect, as compared to control tumors (Figure 5A). To evaluate changes in the gene expression program in these tumors, qPCR analyses were carried out on a selection of representative mesenchymal markers whose up-regulation was previously observed in cells deficient for IRE1α signaling [21]. Glioblastomas expressing IRE1α variants displayed an up-regulated set of genes encoding matrix proteins involved in invasion (Figure 5B). Collagens (COL1A1, COL3A1 and COL5A1) and the collagen cross-linker lysyl oxidase (LOX) transcripts were robustly increased in the presence of doxycycline. In addition, both U87-Y892 and U87-K907-derived tumors displayed overexpression of decorin (DCN), laminins (LAMA1, LAMC1) and thrombospondin-1 (THBS1). This effect was less marked in U87-K599A-derived tumors, which may relate to the attenuated invasive phenotype.

To further address the extent of mesenchymal reprogramming in these tumors, immunofluorescence analysis was used to detect YKL-40 (CHI3L1), a marker of the mesenchymal subtype of glioblastoma and an indicator of cell differentiation toward an invasive phenotype [22, 23]. Consistently, elevated YKL-40 immunoreactivity was observed in glioblastomas expressing IRE1α variants (Figure 5C, upper panel), except for few U87-K599A-derived tumors. The fluorescent signal in U87-Y892A-derived tumors in the presence of doxycycline was about twice higher than in control tumors and a 20% increase was observed in U87-K907A-derived tumors (Figure 5C, lower panel). Again, the intensity of the labeling on U87-K599A-derived tumors was more heterogeneous and was not found statistically different from control tumors.

We next evaluated the intrinsic capacity of cells deficient for IRE1α RNase activity to migrate by examining their chemotactic response under conditions that simulated ischemia. Migration of individual cells under a gradient of serum/glucose was recorded in chemotaxis chambers for 12 h using time-lapse video-microscopy (Figure 5D). Consistent with their invasive behavior *in vivo*, cells expressing IRE1α-Y892A and IRE1α-K907A variants were endowed with a better ability to move toward higher concentrations of serum/glucose. Indeed, a ∼50% increase in their migration rate was measured in the presence of doxycycline relative to condition without doxycycline (Y892A: 1.37 *vs*. 0.92 μm/min; K907A: 1.45 *vs*. 0.95 μm/min; *P* < 0.001). In comparison, doxycycline had no effect on U87-LUC control cells. Finally, the velocity was significantly enhanced in wild-type U87-MG cells treated with the chemical compound 4 μ8C, an inhibitor of both IRE1α RNase and kinase activities [28] (Figure 5E). These results indicated that invalidation of IRE1α RNase induced cell differentiation towards a mesenchymal profile and increased their motility.

### Expression of catalytically active IRE1α C-terminal deletion mutants does not remodel the wild-type phenotype

**Figure 6:**
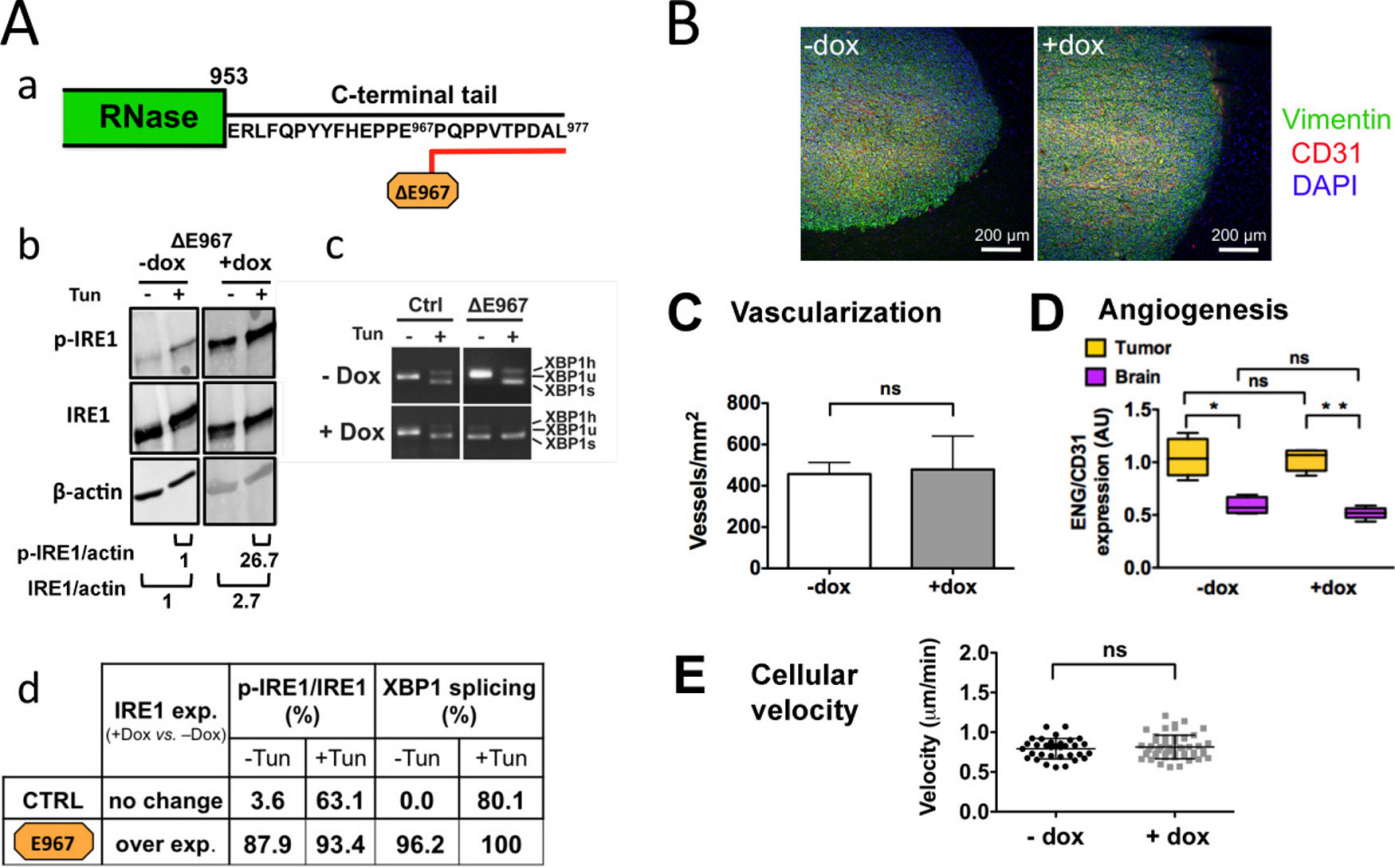
Expression of a catalytically active IRE1α C-terminal deletion mutant does not interfere with the angiogenic and invasive processes Cells were designed to stably express an IRE1α protein truncated at its C-terminus by 10 aminoacids. **Aa.** C-terminal tail deletion of IRE1α; desing of the ΔE967 mutant. Cells were grown for 7 days in culture with or without doxycycline (+Dox and –Dox, respectively) and were analyzed for transgene expression and inhibition of XBP1 mRNA splicing following incubation for 2 h with or without tunicamycine (Tun). **Ab.** Western blot analysis. Quantification of p-IRE1α and total IRE1α proteins were normalized to actin. **Ac.** Measure of XBP1-splicing. **Ad.** Measures in percent (+Dox *vs*. –Dox) of IRE1α kinase and RNase activities in cells expressing either the wild-type IRE1α protein (CTRL) or the IRE1α-ΔE967 transgene product. Relative values of IRE1α autophosphorylation and of XBP1 splicing were determined as in figure 1. **B.** Coronal sections of U87-ΔE967 gliomas grown for 28 days in the mouse brain in the presence or absence of doxycycline. IHC labeling of vimentin, CD31 and DAPI was carried out as in figure 3. **C-D.** Vascularization of glioma tissues and of the brain parenchyma was expressed as the total number of blood vessels (CD31 labeling). Angiogenesis was quantified by measuring the number of proliferative blood vessels (ENG *vs*. CD31 labelings; see figure 3.) **E.** Expression of the IRE1α ΔE967 mutant did not increase cell motility and chemotaxis (see also figure 5D–5E).

The C-terminal tail sequence of human IRE1α protein is predicted to be located apart from the RNase domain [5, 6]. This peptide segment bears putative phosphotyrosine- and PDZ domain-binding groups (http://scansite3.mit.edu/) and we therefore questioned its possible contribution to the neovascularization and invasive processes. If relevant, deletion of this segment would be expected to negatively interfere with the angiogenic features observed in our model. An IRE1α mutant was thus generated exhibiting a 10 aminoacids deletion in its C-terminus, from the glutamate residue at position 967 to the end (IRE1α-ΔE967 mutant) (Figure 6A). Overexpression of this mutant in glioblastoma cells generated constitutive IRE1α autophosphorylation and splicing of XBP1 mRNA, both events being observed in basal condition and under tunicamycin treatment (Figure 6Ab–6Ad). Xenotransplantation of U87 cells expressing this construct developed into massive, vascularized and non-invasive glioblastomas that closely resemble control tumors (Figure 6B) and IRE1wt overexpressing tumors (Figure S2); no significant difference was detected in term of vascularization pattern and blood vessel proliferation (see Figure 6C–6D). Similar results were obtained following ectopic expression of an IRE1α-ΔP965 mutant truncated at the C-terminus by 12 aminoacids (not shown). Moreover, the property of ΔE967 tumor cells to migrate along a nutrient gradient was not significantly modified (Figure 6E). Thus, the C-term sequence did not contribute significantly to glioma vascularization and invasion. Notably, permanent activation of both enzymes did not reshape the wild-type tumor phenotype.

## DISCUSSION

High-grade gliomas represent the most common primary brain neoplasms in human and are associated with a poor clinical outcome [25]. Important efforts have therefore been developed in order to better understand the pivotal roles of pathological angiogenesis and invasiveness in this malignancy. Intense microvascular proliferation correlates with higher histologic grades of gliomas [25], an observation which has led to the development of efficient anti-angiogenic strategies aimed at blocking the disease progression. However, these treatments may also stimulate the invasive growth behavior of glioblastoma cells, thereby leading to relapses with diffuse patterns and perivascular growth [24, 26]. The cellular and molecular mechanisms underlying these distinct effects are not well understood.

IRE1α, the most conserved stress sensor of the UPR, is a single-pass transmembrane protein of the ER. Under cellular stress, allosteric triggering of IRE1α leads to its trans-autophosphorylation and RNase activation [2, 3, 6, 7]. IRE1α downstream signal transduction then involves a set of distinct mechanisms including *i)* activation of JNK and NF-κB, *ii)* IRE1α RNase-mediated RNA decay (RIDD) of RNA and *iii)* unconventional splicing of XBP1 mRNA [1, 2, 9, 10, 14, 15].

The role of IRE1α signaling in cancer progression remains to be fully clarified. The IRE1α/XBP1 transduction axis clearly contributes in tumor development [13, 16, 17], which is consistent with its reported role in mediating the adaptive response to ischemia [29]. Such a beneficial response to stress may however adversely support tumor resistance to chemotherapies [30, 31]. To this end, both pro- and anti-neoplastic properties of the ER-stress are being currently considered for new therapeutic avenues [2, 30, 31]. As such, functional blockade of IRE1α produced ambivalent antitumor effects in a glioblastoma xenograft model by inhibiting angiogenesis and concurrently inducing cancer cell migration [18, 21].

In order to evaluate more precisely the implication of IRE1α in these distinct outcomes, we examined in this work the effects of single amino-acid substitutions in IRE1α catalytic domains on glioblastoma development.To this end, one mutant was designed to invalidate IRE1α kinase activity (K599A) whereas two others (Y892A and K907A) targeted IRE1α RNase domain.

As expected, expression of the Y982A RNase mutant abolished XBP1 splicing under ER stress. The protein variant also exhibited an elevated rate of autophosphorylation, consistent with its high level of expression [8, 32]. *In vivo*, expression of the Y892A mutant constrained glioblastoma progression but did not impede the formation of new blood vessels, whose general architecture was not different from that of control tumors. However, as compared with massive and well-delineated U87 control tumor bulks, glioblastomas expressing the IRE1α-Y892A mutant were invariably diffuse and harbored two distinct features of invasion: a short-range infiltration at the immediate periphery of glioblastoma cores, and an extensive blood vessel co-option with formation of distal perivascular tumor microsatellites. These two modes of dissemination were also recognized within the core of IRE1α-Y892A gliomas, therefore suggesting that they might reflect an intrinsic property of tumor cells rather than a reaction to the micro-environmental selection pressure. Thus, blockade of the IRE1α RNase induced a major phenotypic switch towards a pro-invasive mode.

The dual phenotype of IRE1α-Y892A glioblastomas indicates that the formation of new blood vessels does not preclude cancer cell dissemination or co-option. Such a mixed phenotype has been reported in other preclinical models [33], and high-grade glioma cells of mesenchymal subtype also elicit both infiltrative and angiogenic characters [22, 23]. Similarly, invasive/co-optive IRE1α dominant-negative glioblastomas concomitantly exhibited a highly vascularized pattern when producing ectopic interleukin-6 [21].

Expression of the second RNase mutant K907A resulted in glioblastoma cells that are essentially deficient for IRE1α. *In vivo*, this mutant recapitulated the main characteristics obtained by using either IRE1α silencing or IRE1 dominant-negative constructs [21]: avascular and infiltrative tumors were obtained and the growth rate of malignant cells was dramatically lower. Hence, both U87-Y892A and U87-K907A cells promoted the development of infiltrative glioblastomas and also exhibited a higher velocity *in vitro*. This result is in agreement with the higher potential of wild-type tumor cells to migrate in culture in the presence of 4 μ8C, a small-molecule IRE1α inhibitor.

Similar analyses were also performed with the K599A mutant. In our experimental conditions, a low level of auto-phosphorylation was still observed (see also ref. [5]). Likewise, a minor splicing of XBP1 was detected upon tunicamycin treatment in ∼20% of our assays, which correlates with the low RNase activity detected in cells expressing the same mutant under thapsigargin treatment [34]. These effects presumably resulted from the endogenous activity of the wild-type IRE1α protein, although the existence of positive crosstalks from cytoplasmic kinases to IRE1α could not be excluded [35, 36]. The fact that remnant functions of IRE1α still exist in cells expressing the IRE1α-K599A mutant is also consistent with *in vitro* experiments showing that dephosphorylated or kinase dead IRE1α from yeast or human still exhibited low RNase activity following oligomerization [8, 32]. This residual IRE1α activity provided a rationale to the fact that glioblastomas expressing IRE1α-K599A mutant exhibited moderate infiltrative features: diffuse lesions had lower penetrance (∼60%), grew less compact than control tumors and no co-option was observed. Besides, the vascularization pattern in these tumors was similar to that observed in wild-type tumors.

Invasiveness was shown to correlate with the overexpression of matrix proteins in the mesenchymal glioma subtype [22, 23]. Since abrogation of the IRE1α RNase activity reprograms cells towards a motile behavior, we therefore considered the production of matrix proteins in this glioma model. Clearly, cells expressing IRE1α RNase mutants heightened the expression of ECM markers characteristic of glioblastoma malignancy or invasion, including fibrillar collagens, the collagen cross-linker LOX and the proteins THBS1, YKL-40, DCN and LAMA4 [22, 23, 37]. These results are consistent with the mesenchymal gene expression signature associated to U87 cells in culture or in preclinical models, either after blockade of IRE1α function [21] or following treatments with anti-angiogenic agents [38, 39]. This suggests that the inhibition of signaling events downstream of IRE1α RNase activity, involving the cytosolic RNA decay (RIDD) and/or XBP1 mRNA splicing [9, 12], might contribute to mesenchymal transformation. The inhibition of IRE1α-mediated cleavage of mRNAs encoding the invasion marker SPARC and other ECM candidate targets [9, 18], as well as of microRNAs [40] functionally associated to tumor invasiveness [41–43], could participate to this phenotypic switch.

The functional analysis of IRE1α mutants in this glioblastoma model provides evidence of two distinct contributions of IRE1α in pathological invasion and angiogenesis, respectively. Indeed, invalidation of the RNase activity was sufficient for triggering invasiveness. In addition, our data suggest a permissive role of IRE1α, but not of its RNAse activity, in glioma neovascularization. IRE1α kinase activity was found to express selective functions in animals and plants [14, 15, 44, 45] and therefore represents an attractive candidate for regulating blood vessel formation. Indeed, in the absence of its RNase domain, the IRE1α cytosolic moiety still has the ability to recruit the adaptor protein TRAF-2, which represents a potential contributor to angiogenesis through activation of JNK and NF-kB-dependent signaling pathways [14, 15]. How this system is modulated *in vivo*, however, remains an open question.

Finally, it should be emphasized that glioblastomas expressing any of the IRE1α mutations reported here developed at a slower rate than the control lesions, the overall survival of implanted animals being consistently prolonged in each case. We showed that apoptosis was not the primary cause of the decrease in tumor growth. This effect resulted at least in part of a lower mitotic index, which agreed well with the positive correlation reported between the activity of the IRE1α/XBP1 branch and tumor cell proliferation [16, 21, 29, 34].

In conclusion, our results suggest that IRE1α is a major molecular integrator of angiogenesis and mesenchymal differentiation under the control of biological and chemical stimuli. From the clinical perspective, an appealing anti-tumor combination would be to maintain IRE1α RNase activity (anti-invasive effect) while blocking kinase functions (anti-angiogenic effect). Interestingly, such a prospect was recently substantiated *in vitro* by using either ATP-competitive small molecules inhibitors [3, 46] or invalidation mutants of the protein kinase active site [47] that potently activate IRE1α endoribonuclease. Nevertheless, the relevance of such approaches has to be appreciated in a biological context, considering the fact that the IRE1α/XBP1 branch was also reported to signal angiogenesis in other malignancies [13]. Understanding and delineating IRE1α-dependent downstream pathways involved in tumor cell infiltration and blood vessel proliferation should help to consider new therapeutic opportunities for the treatment of malignant gliomas.

## MATERIALS AND METHODS

### Reagents

Culture media were from Life Technologies. Antibodies were as previously reported [21]. Others antibodies were obtained as follows: vinculin and FLAG M2 (Sigma); NG2 (Millipore); phospho-(Ser51)-eIF2α and eIF2α (Cell Signaling); YKL-40 (Quidel). Doxycycline hyclate was from Sigma. Primers were as indicated in Table S1.

### Site directed mutagenesis and expression of IRE1α mutants

Human IRE1α cDNA was obtained from the plasmid pEDhIRE1 kindly provided by R. Kaufman. The residues lysine-599, tyrosine-892 and lysine-907 were substituted by alanine using the Quickchange II XL site-directed mutagenesis kit (Agilent). Mutants truncated in the C-terminal segment of IRE1 (proline-965 and glutamate-967) were generated by introducing a stop codon in the IRE1α ORF at these positions. A HindIII/XhoI fragment of pcDNA3.1-hIRE1 was replaced with the homologous mutated fragments. IRE1α-mutated sequences were confirmed by sequencing and subcloned into the lentiviral vector pTRIP-DU3-TRETight (Genomic Platform, Université P. Sabatier, Toulouse, France) to obtain pTRIP-hIRE1 -K599A, -Y892A, -K907A, -ΔP965 and -ΔE967 constructs. The Tet-ON empty vector and the same vector expressing GFP (pTRIP-GFP) were also used. Vectors were transduced in a polyclonal U87-MG cell population expressing luciferase (U87-LUC cells). Proteins were expressed under the control of the doxycycline promoter.

### Cell culture

Cells were grown in DMEM, 4.5 g/L glucose supplemented with 10% FBS, L-glutamine, and antibiotics. Gene expression under the dependence of the Tet-ON promoter was induced in culture by adding 2 μg/ml doxycycline for two days (K599A, Y892A and K907A mutants) or for seven days (ΔP965 and ΔE967 mutants).

### ATF6α activation assay

Measure of ATF6α activation was performed as previously reported [48]. Cells were transiently transfected with FLAG-ATF6α and incubated for 24 h with or without 2 μg/ml doxycycline. U87 control cells over-expressing IRE1α wild-type protein (IRE1wt) or bearing the empty vector (EV) were analyzed following incubation for 3 h with or without DTT. FLAG-ATF6α protein and calnexin (CNX) were revealed simultaneously by using anti-FLAG mouse antibodies and anti-CNX rabbit antibodies, respectively, followed by incubation with Alexa Fluor 488-labeled anti-mouse IgG and Alexa Fluor 568-labeled anti-rabbit IgG secondary antibodies.

### Intracranial implantations

Glioma cells (5 × 10^4^ cells, 1 μl) were implanted in 8–9 weeks of age RAG2/γ_c_ mice [21]. Mice were randomized and fed either with drinking water only (−Dox) or with water containing 1 mg/ml doxycycline (+Dox) from day 3 after implantation until the end of experimentations. Kaplan–Meier analysis was used for survival analysis [21]. Experiments were performed at the animal facility, Université de Bordeaux, according to the ethical criteria approved by the Ministère de l'Enseignement Supérieur et de la Recherche (MESR).

### Immunoblot and PCR analyses

IRE1α and phospho-(Ser724)-IRE1α proteins were revealed by immunobloting as described previously [21]. RNA extraction, quantification and gene expression analyses were as reported [21]. RT-qPCR results were the mean of triplicate determinations ± SD for each glioblastoma sample (*n* = 5 mice per condition). XBP1 splicing was quantified according to Shang and Lehrman [49].

### Real-time imaging

Mice at days 17, 27 and 37 post-implantation were anesthetized with 2% isoflurane, injected intraperitoneally with 150 mg/kg D-luciferin (Promega) and placed in a photonIMAGER™ chamber (Biospace Lab). White light and luciferase activity images were monitored at 30 sec intervals for 5 min and images were analyzed using the Living Image software M3 Vision (Biospace Lab). Signal intensity was quantified as the sum of all detected photon counts from tumors. Signal average for each group of mice (+Dox) was compared with those from control animals (−Dox). Apoptosis was monitored one day before tumor growth imaging. Mice were injected *i.p*. with VivoGlo™ Caspase-3/7 substrate (50 mg/kg; Promega) and subjected to a 5 min imaging session. Acquisition of the Luciferase apoptotic signal and ROI measurement were then performed. Z-DEVD-aminoluciferin signal was normalized with the D-luciferin signal. Signal average for each group was calculated and results were analyzed for statistical significance using a Mann-Whitney *t*-test. Values are the mean ± SD.

### Analysis of glioma phenotypes

Immunohistochemical and immunofluorescence analyses were performed on cryosections as previously reported [21]. Imaging was carried out by using a Nikon eclipse E600 microscope or confocal SP8 LEICA and Nikon N-SIM microscopes. YKL-40 labeling was quantified by using the ImageJ software. At least five brains were analyzed for each condition.

### Cell chemotaxis

Chemotaxis measurement in real-time live-cell imaging was carried out in 10 μ-Slide Chemotaxis^3D^ plates (#80322; Ibidi Biovalley) according to manufacturer's instructions. Cells grown in culture for three days with or without 2 μg/ml doxycycline were then incubated (3.6 × 10^4^ cells) for 25 min in chemotaxis plates in DMEM containing 1.5 mg/ml of collagen I without serum and glucose. Microplates were then filled with DMEM medium with or without 10% FBS, 1 mg/ml glucose and 2 μg/ml doxycycline. Time-lapse microscopy was carried out in a humidified 5% CO_2_ atmosphere at 37°C using a Zeiss observer Z1 videomicroscope (Carl Zeiss) and photomicrographs were taken at constant intervals of 15 min for 12 h. Four to six different fields were recorded for each condition using the AxioVision Rel 4.8 software. Quantitative image analysis of cell motility tracking was processed using the ImageJ software (25 ≤ *n* ≤ 50; cells per field). Statistical significance was evaluated by using a non-parametric *t*-test for comparison between different culture conditions in a single mutant, and by one-way ANOVA when comparing each pair of mutants.

## SUPPLEMENTARY FIGURES AND TABLE



## References

[1] Hetz C, Glimcher LH (2009). Fine-tuning of the unfolded protein response: Assembling the IRE1alpha interactome. Mol Cell.

[2] Walter P, Ron D (2011). The unfolded protein response: from stress pathway to homeostatic regulation. Science.

[3] Han D, Lerner AG, Vande Walle L, Upton JP, Xu W, Hagen A, Backes BJ, Oakes SA, Papa FR (2009). IRE1alpha kinase activation modes control alternate endoribonuclease outputs to determine divergent cell fates. Cell.

[4] Tirasophon W, Lee K, Callaghan B, Welihinda A, Kaufman RJ (2000). The endoribonuclease activity of mammalian IRE1 autoregulates its mRNA and is required for the unfolded protein response. Genes Dev.

[5] Tirasophon W, Welihinda AA, Kaufman RJ (1998). A stress response pathway from the endoplasmic reticulum to the nucleus requires a novel bifunctional protein kinase/endoribonuclease (Ire1p) in mammalian cells. Genes Dev.

[6] Lee KP, Dey M, Neculai D, Cao C, Dever TE, Sicheri F (2008). Structure of the dual enzyme Ire1 reveals the basis for catalysis and regulation in nonconventional RNA splicing. Cell.

[7] Ali MM, Bagratuni T, Davenport EL, Nowak PR, Silva-Santisteban MC, Hardcastle A, McAndrews C, Rowlands MG, Morgan GJ, Aherne W, Collins I, Davies FE, Pearl LH (2011). Structure of the Ire1 autophosphorylation complex and implications for the unfolded protein response. Embo J.

[8] Korennykh AV, Egea PF, Korostelev AA, Finer-Moore J, Zhang C, Shokat KM, Stroud RM, Walter P (2009). The unfolded protein response signals through high-order assembly of Ire1. Nature.

[9] Hollien J, Lin JH, Li H, Stevens N, Walter P, Weissman JS (2009). Regulated Ire1-dependent decay of messenger RNAs in mammalian cells. J Cell Biol.

[10] Maurel M, Chevet E, Tavernier J, Gerlo S (2014). Getting RIDD of RNA: IRE1 in cell fate regulation. Trends Biochem Sci.

[11] Yoshida H, Matsui T, Yamamoto A, Okada T, Mori K (2001). XBP1 mRNA is induced by ATF6 and spliced by IRE1 in response to ER stress to produce a highly active transcription factor. Cell.

[12] Acosta-Alvear D, Zhou Y, Blais A, Tsikitis M, Lents NH, Arias C, Lennon CJ, Kluger Y, Dynlacht BD (2007). XBP1 controls diverse cell type- and condition-specific transcriptional regulatory networks. Mol Cell.

[13] Chen X, Iliopoulos D, Zhang Q, Tang Q, Greenblatt MB, Hatziapostolou M, Lim E, Tam WL, Ni M, Chen Y, Mai J, Shen H, Hu DZ (2014). XBP1 promotes triple-negative breast cancer by controlling the HIF1alpha pathway. Nature.

[14] Urano F, Wang X, Bertolotti A, Zhang Y, Chung P, Harding HP, Ron D (2000). Coupling of stress in the ER to activation of JNK protein kinases by transmembrane protein kinase IRE1. Science.

[15] Hu P, Han Z, Couvillon AD, Kaufman RJ, Exton JH (2006). Autocrine tumor necrosis factor alpha links endoplasmic reticulum stress to the membrane death receptor pathway through IRE1alpha-mediated NF-kappaB activation and down-regulation of TRAF2 expression. Mol Cell Biol.

[16] Carrasco DR, Sukhdeo K, Protopopova M, Sinha R, Enos M, Carrasco DE, Zheng M, Mani M, Henderson J, Pinkus GS, Munshi N, Horner J, Ivanova EV (2007). The differentiation and stress response factor XBP-1 drives multiple myeloma pathogenesis. Cancer Cell.

[17] Romero-Ramirez L, Cao H, Nelson D, Hammond E, Lee AH, Yoshida H, Mori K, Glimcher LH, Denko NC, Giaccia AJ, Le QT, Koong AC (2004). XBP1 is essential for survival under hypoxic conditions and is required for tumor growth. Cancer Res.

[18] Dejeans N, Pluquet O, Lhomond S, Grise F, Bouchecareilh M, Juin A, Meynard-Cadars M, Bidaud-Meynard A, Gentil C, Moreau V, Saltel F, Chevet E (2012). Autocrine control of glioma cells adhesion and migration through IRE1alpha-mediated cleavage of SPARC mRNA. J Cell Sci.

[19] Pluquet O, Dejeans N, Bouchecareilh M, Lhomond S, Pineau R, Higa A, Delugin M, Combe C, Loriot S, Cubel G, Dugot-Senant N, Vital A, Loiseau H (2013). Posttranscriptional regulation of PER1 underlies the oncogenic function of IREalpha. Cancer Res.

[20] Greenman C, Stephens P, Smith R, Dalgliesh GL, Hunter C, Bignell G, Davies H, Teague J, Butler A, Stevens C, Edkins S, O'Meara S, Vastrik I (2007). Patterns of somatic mutation in human cancer genomes. Nature.

[21] Auf G, Jabouille A, Guerit S, Pineau R, Delugin M, Bouchecareilh M, Magnin N, Favereaux A, Maitre M, Gaiser T, von Deimling A, Czabanka M, Vajkoczy P (2010). Inositol-requiring enzyme 1alpha is a key regulator of angiogenesis and invasion in malignant glioma. Proc Natl Acad Sci U S A.

[22] Phillips HS, Kharbanda S, Chen R, Forrest WF, Soriano RH, Wu TD, Misra A, Nigro JM, Colman H, Soroceanu L, Williams PM, Modrusan Z, Feuerstein BG (2006). Molecular subclasses of high-grade glioma predict prognosis, delineate a pattern of disease progression, and resemble stages in neurogenesis. Cancer Cell.

[23] Carro MS, Lim WK, Alvarez MJ, Bollo RJ, Zhao X, Snyder EY, Sulman EP, Anne SL, Doetsch F, Colman H, Lasorella A, Aldape K, Califano A (2010). The transcriptional network for mesenchymal transformation of brain tumours. Nature.

[24] Lu KV, Bergers G (2013). Mechanisms of evasive resistance to anti-VEGF therapy in glioblastoma. CNS Oncol.

[25] Kleihues P, Burger PC, Aldape KD, Brat DJ, Biernat W, Bigner DD, Nakazato Y, Plate KH, Giangaspero F, von Deimling A, Ohgaki H, Cavenee WH (2007). WHO Classification of Tumours of the Central Nervous System.

[26] Verhoeff JJ, van Tellingen O, Claes A, Stalpers LJ, van Linde ME, Richel DJ, Leenders WP, van Furth WR (2009). Concerns about anti-angiogenic treatment in patients with glioblastoma multiforme. BMC Cancer.

[27] Chekenya M, Enger PO, Thorsen F, Tysnes BB, Al-Sarraj S, Read TA, Furmanek T, Mahesparan R, Levine JM, Butt AM, Pilkington GJ, Bjerkvig R (2002). The glial precursor proteoglycan, NG2, is expressed on tumour neovasculature by vascular pericytes in human malignant brain tumours. Neuropathol Appl Neurobiol.

[28] Cross BC, Bond PJ, Sadowski PG, Jha BK, Zak J, Goodman JM, Silverman RH, Neubert TA, Baxendale IR, Ron D, Harding HP (2012). The molecular basis for selective inhibition of unconventional mRNA splicing by an IRE1-binding small molecule. Proc Natl Acad Sci U S A.

[29] Spiotto MT, Banh A, Papandreou I, Cao H, Galvez MG, Gurtner GC, Denko NC, Le QT, Koong AC (2010). Imaging the unfolded protein response in primary tumors reveals microenvironments with metabolic variations that predict tumor growth. Cancer Res.

[30] Epple LM, Dodd RD, Merz AL, Dechkovskaia AM, Herring M, Winston BA, Lencioni AM, Russell RL, Madsen H, Nega M, Dusto NL, White J, Bigner DD (2013). Induction of the unfolded protein response drives enhanced metabolism and chemoresistance in glioma cells. PLoS One.

[31] Hetz C, Chevet E, Harding HP (2013). Targeting the unfolded protein response in disease. Nat Rev Drug Discov.

[32] Itzhak D, Bright M, McAndrew P, Mirza A, Newbatt Y, Strover J, Widya M, Thompson A, Morgan G, Collins I, Davies F (2014). Multiple autophosphorylations significantly enhance the endoribonuclease activity of human inositol requiring enzyme 1alpha. BMC Biochem.

[33] Holash J, Maisonpierre PC, Compton D, Boland P, Alexander CR, Zagzag D, Yancopoulos GD, Wiegand SJ (1999). Vessel cooption, regression, and growth in tumors mediated by angiopoietins and VEGF. Science.

[34] Thorpe JA, Schwarze SR (2010). IRE1alpha controls cyclin A1 expression and promotes cell proliferation through XBP-1. Cell Stress Chaperones.

[35] Mao T, Shao M, Qiu Y, Huang J, Zhang Y, Song B, Wang Q, Jiang L, Liu Y, Han JD, Cao P, Li J, Gao X (2011). PKA phosphorylation couples hepatic inositol-requiring enzyme 1alpha to glucagon signaling in glucose metabolism. Proc Natl Acad Sci U S A.

[36] Zeng L, Xiao Q, Chen M, Margariti A, Martin D, Ivetic A, Xu H, Mason J, Wang W, Cockerill G, Mori K, Li JY, Chien S (2013). Vascular endothelial cell growth-activated XBP1 splicing in endothelial cells is crucial for angiogenesis. Circulation.

[37] Freije WA, Castro-Vargas FE, Fang Z, Horvath S, Cloughesy T, Liau LM, Mischel PS, Nelson SF (2004). Gene expression profiling of gliomas strongly predicts survival. Cancer Res.

[38] Saidi A, Hagedorn M, Allain N, Verpelli C, Sala C, Bello L, Bikfalvi A, Javerzat S (2009). Combined targeting of interleukin-6 and vascular endothelial growth factor potently inhibits glioma growth and invasiveness. Int J Cancer.

[39] Piao Y, Liang J, Holmes L, Henry V, Sulman E, de Groot JF (2013). Acquired resistance to anti-VEGF therapy in glioblastoma is associated with a mesenchymal transition. Clin Cancer Res.

[40] Upton JP, Wang L, Han D, Wang ES, Huskey NE, Lim L, Truitt M, McManus MT, Ruggero D, Goga A, Papa FR, Oakes SA (2012). IRE1alpha cleaves select microRNAs during ER stress to derepress translation of proapoptotic Caspase-2. Science.

[41] Guessous F, Zhang Y, Kofman A, Catania A, Li Y, Schiff D, Purow B, Abounader R (2010). microRNA-34a is tumor suppressive in brain tumors and glioma stem cells. Cell Cycle.

[42] Moller HG, Rasmussen AP, Andersen HH, Johnsen KB, Henriksen M, Duroux M (2013). A systematic review of microRNA in glioblastoma multiforme: micro-modulators in the mesenchymal mode of migration and invasion. Mol Neurobiol.

[43] Siemens H, Jackstadt R, Hunten S, Kaller M, Menssen A, Gotz U, Hermeking H (2011). miR-34 and SNAIL form a double-negative feedback loop to regulate epithelial-mesenchymal transitions. Cell Cycle.

[44] Tam AB, Mercado EL, Hoffmann A, Niwa M (2012). ER stress activates NF-kappaB by integrating functions of basal IKK activity, IRE1 and PERK. PLoS One.

[45] Wakasa Y, Hayashi S, Ozawa K, Takaiwa F (2012). Multiple roles of the ER stress sensor IRE1 demonstrated by gene targeting in rice. Sci Rep.

[46] Wang L, Perera BG, Hari SB, Bhhatarai B, Backes BJ, Seeliger MA, Schurer SC, Oakes SA, Papa FR, Maly DJ (2012). Divergent allosteric control of the IRE1alpha endoribonuclease using kinase inhibitors. Nat Chem Biol.

[47] Rubio C, Pincus D, Korennykh A, Schuck S, El-Samad H, Walter P (2011). Homeostatic adaptation to endoplasmic reticulum stress depends on Ire1 kinase activity. J Cell Biol.

[48] Higa A, Taouji S, Lhomond S, Jensen D, Fernandez-Zapico ME, Simpson JC, Pasquet JM, Schekman R, Chevet E (2014). Endoplasmic reticulum stress-activated transcription factor ATF6alpha requires the disulfide isomerase PDIA5 to modulate chemoresistance. Mol Cell Biol.

[49] Shang J, Lehrman MA (2004). Discordance of UPR signaling by ATF6 and Ire1p-XBP1 with levels of target transcripts. Biochem Biophys Res Commun.

