# Supplementary material for: Glioblastoma invasion and cooption depend on IRE1α endoribonuclease activity
Source: Oncotarget. 2015 Jul 23;6(28):24922–34. doi: 10.18632/oncotarget.4679 (PMC4694804; doi:10.18632/oncotarget.4679)
Supplement: Supplementary file 1 [file oncotarget-06-24922-s001.pdf]

SUPPLEMENTARY FIGURES AND TABLE

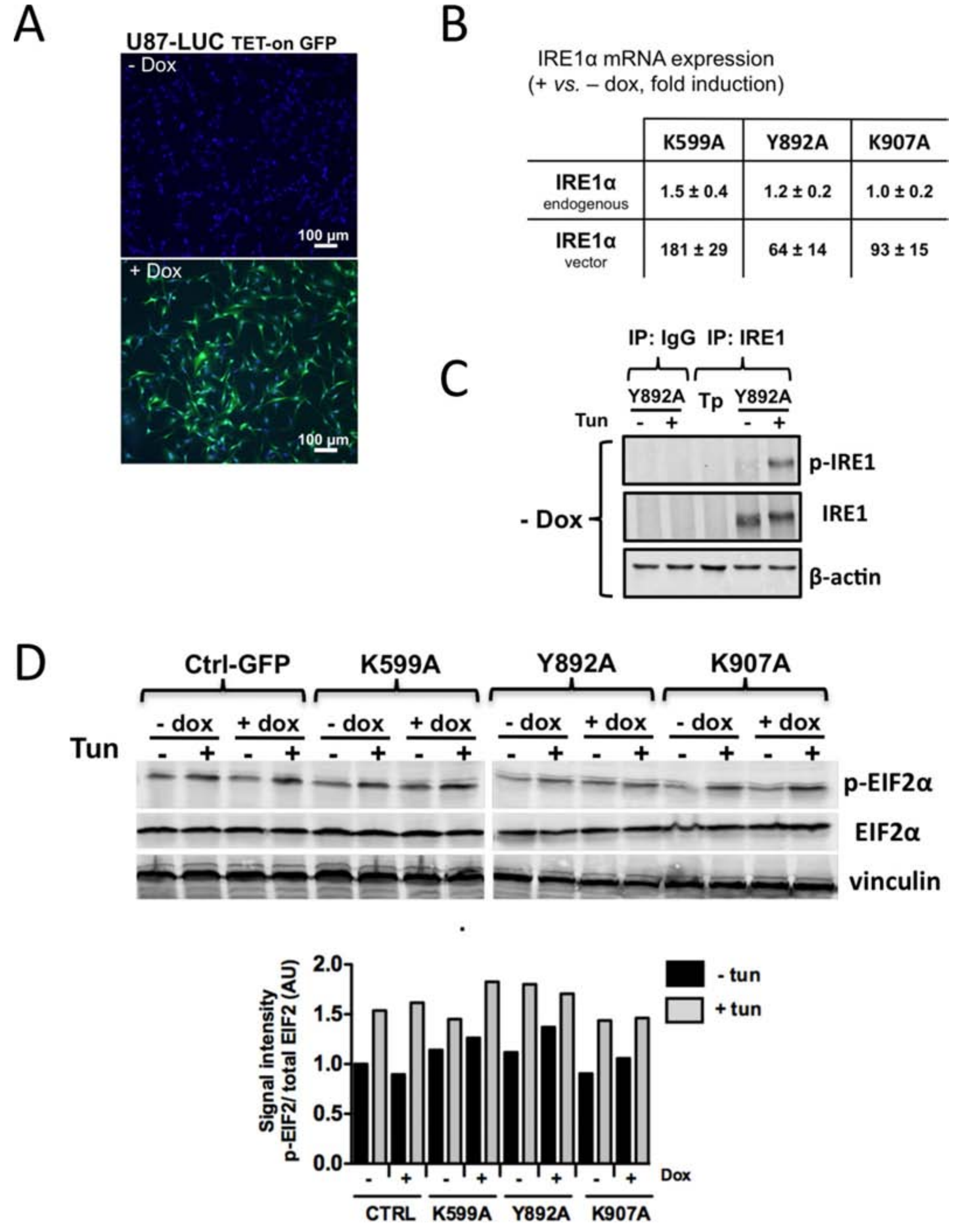

Supplementary Figure S1: Characterization of IRE1α mutants. A. U87-LUC cells were transduced with the lentiviral vector containing the GFP sequence (U87-LUC Tet-ON GFP). Cells were grown for 48 h with (+Dox) or without (-Dox) doxycycline and photographed. **B.** U87-Y892A, U87-K599A and U87-K907A cells were grown for 2 days with or without doxycycline, and the expression of endogenous or ectopic IRE1α transcripts was measured by using RT-PCR and expressed as fold-induction (+) dox *vs.* (−) dox. **C.** U87-Y892A, U87-K599A and U87-K907A cells were grown without doxycycline and were stimulated with or without tunicamycin for 2 h. IRE1α protein was immunoprecipitated from cellular extracts using anti-IRE1α antibodies (IP: IRE1) or using non-specific rabbit IgG (IP: IgG). Same experiment was performed in the absence of antibodies (Tp). Phospho-(Ser724)-IRE1α (p-IRE1), total IRE1α (IRE1) and β-actin were revealed by immunoblotting. **D.** U87 Tet-on GFP, U87-K599A, U87-Y892A and U87-K907A cells were grown in the absence (-dox) or presence (+dox) of doxycycline. They were then stimulated with (+) or without (−) tunicamycin for 2 h and subsequently subjected to an immunoblot by using antibodies against phosphorylated Ser-51 eIF2α (p-EIF2α), total eIF2α or vinculin as internal loading control. Upper panel: Western blot analysis; lower panel: quantification of the ratio p-IRE1α/IRE1α relative to vinculin.

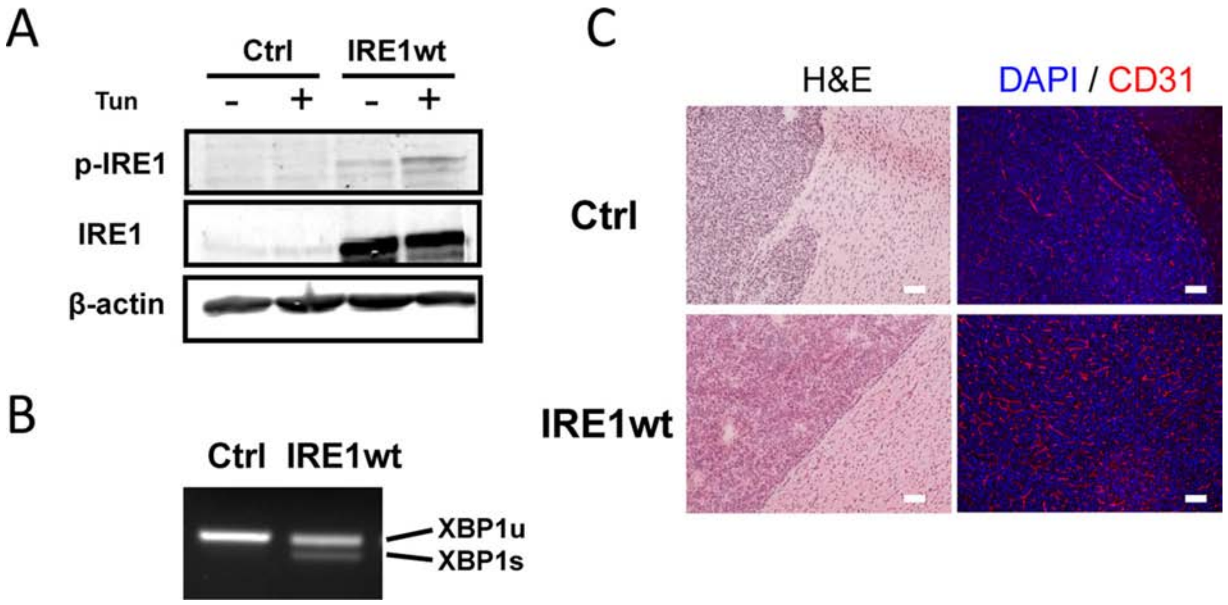

Supplementary Figure S2: Ectopic expression of IRE1wt protein does not affect significantly the angiogenic and non-invasive characteristics of U87 cell-derived gliomas. Control tumor cells and cells over-expressing the IRE1wt protein were implanted intracerebrally in mice. A) High expression of IRE1 in U87 cells after transduction of the wild-type IRE1 (IRE1wt) gene. Western blot analysis of total IRE1 (IRE1) and of phospho-Ser724-IRE1 (p-IRE1). B) XBP1 splicing was increased in U87 cells overexpressing IRE1wt. XBP1u, unspliced mRNA; XBP1s, spliced mRNA. C) H&E staining (left panels) and CD31 labeling (right panels) were carried out on coronal sections of the brain tumors (Ctrl: tumors bearing the empty vector; IRE1wt, tumors over-expressing the IRE1 wild-type protein). Bars: 100 µm.

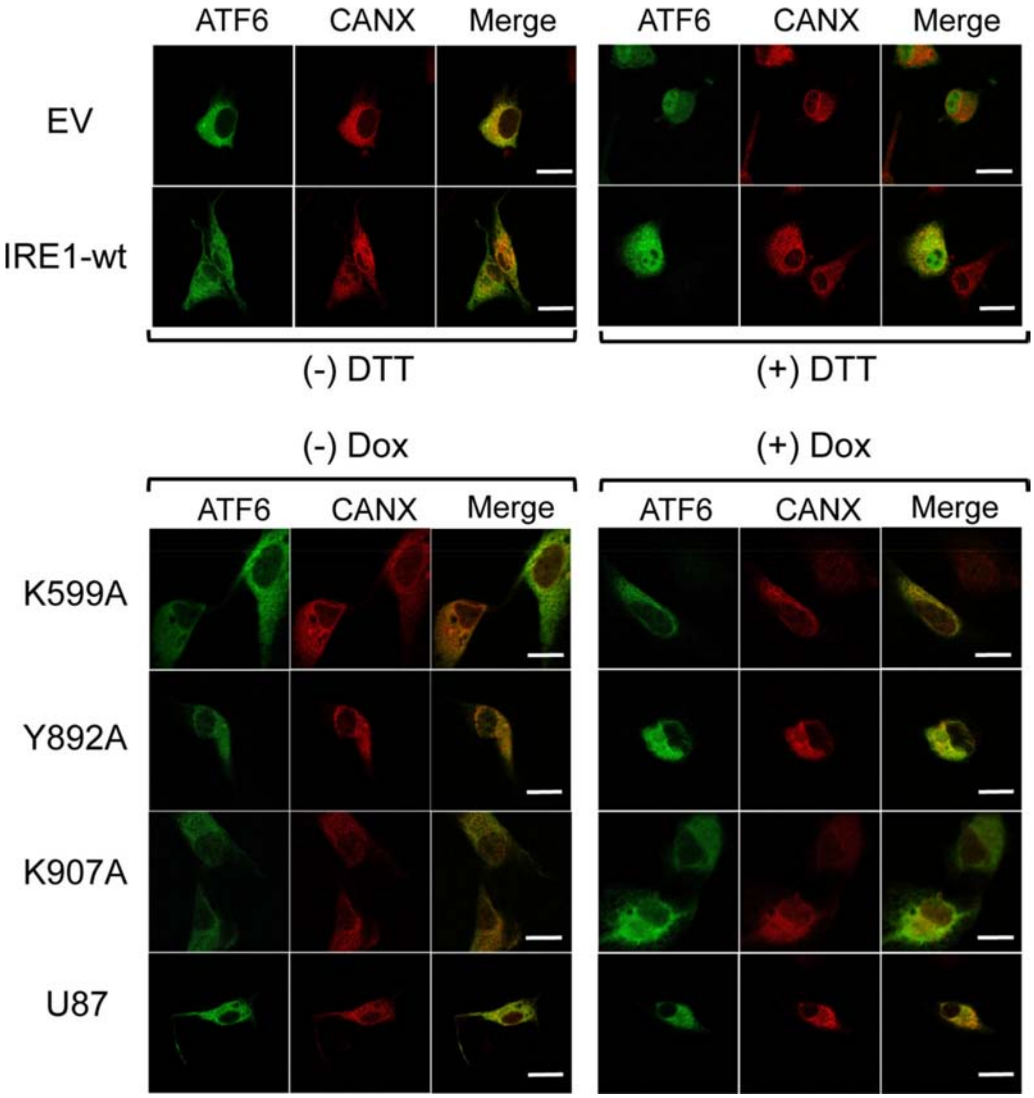

Supplementary Figure S3: ATF6α activation assay. Cells expressing the different IRE1α mutants or the IRE1wt protein were transfected with FLAG-ATF6α plasmid for 24 h and incubated in the presence or abence of doxycycline (Dox) with 1 mM DTT for three hours, as indicated. Cells were then immunostained using anti-FLAG (ATF6α; green labeling) and anti-CANX (endoplasmic reticulum; red labeling) antibodies and were analyzed by confocal microscopy. Bars: 10 µm.

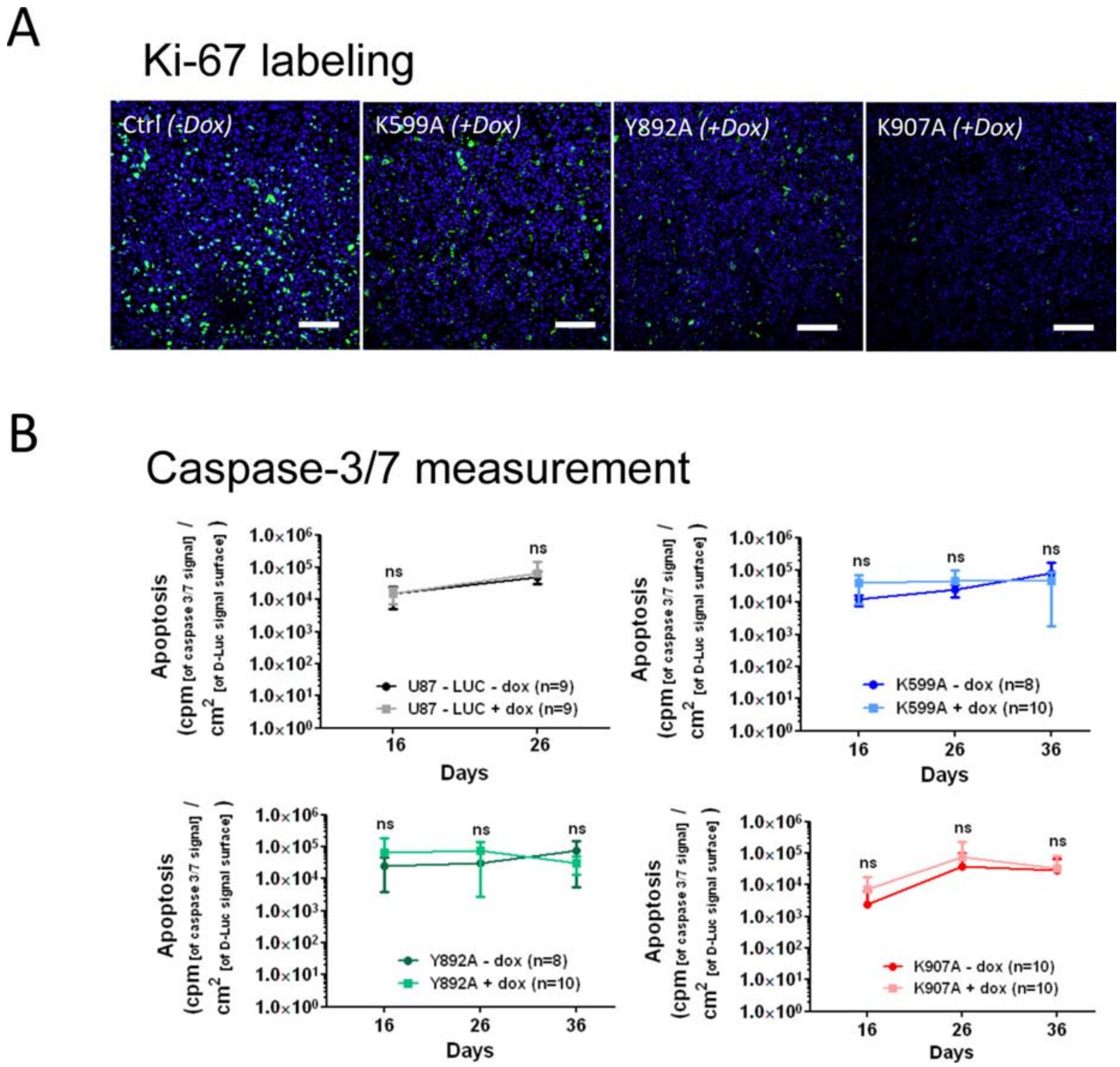

Supplementary Figure S4: Decreased proliferation rate in IRE1α mutants. A. Proliferation index of implanted U87-K599A, U87-Y892A and U87-K907A gliomas with or without doxycycline (see also Figure 3D). Tumors were immunostained using antibodies anti-Ki-67. (Scale bars: 100 μm). **B.** Real-time bioluminescence imaging of apoptosis in mice bearing U87-LUC, U87-K599A, U87-Y892A and U87-K907A gliomas. Apoptosis was monitored after intraperitoneal injection of the caspase-3/7 synthetic substrate (Z-DEVD-aminoluciferin) one day before tumor measurement (injection of D-luciferin; days 16, 26 and 36). Bioluminescence intensity of apoptosis (in cpm) was normalized to that of tumor size (in cm^2). n, number of mice. Statistical analysis represents means values ± SD. (ns, $P > 0.05$).

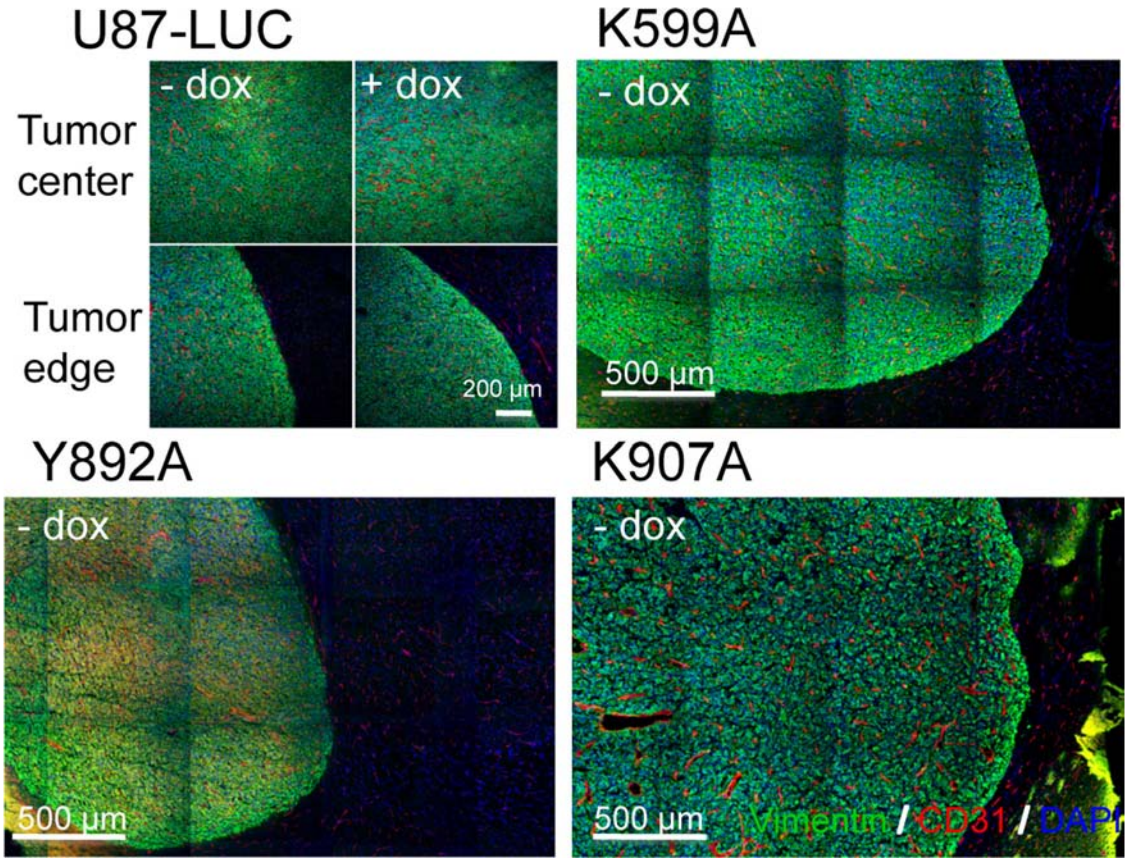

Supplementary Figure S5: Control U87-gliomas are angiogenic and non-infiltrative. Coronal sections were obtained from U87-LUC, U87-K599A, U87-K907A and U87-Y892A tumors in the absence of doxycycline (see also figure 3). Immunolabeling was performed using anti-vimentin (tumor cells) and anti-CD31 (blood vessels) antibodies. Microphotographs are representative of at least 5 brain tumors analyzed for each condition.

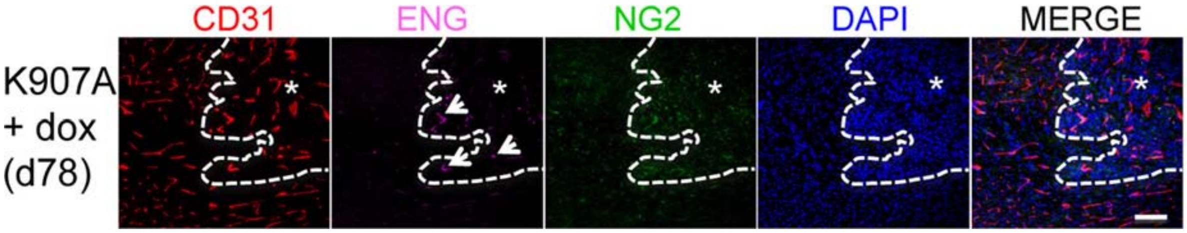

Supplementary Figure S6: Long-lasting avascular phenotype with vessel co-option in gliomas deficient for IRE1α. U87-K907A tumors were grown for 78 days with doxycycline and were immunostained against CD31, ENG and NG2. Arrows point to the presence of rare angiogenic vessels among co-opted vessels into the glioma tissue. Asterisks localize tumor tissue delimited by the dashed line.

Supplementary Table S1. Primers used in this study

gene	forward primer (5’- > 3’)	reverse primer (5’- > 3’)
β-actin	CGTACCACTGGCATCGTGAT	GTGTTGGCGTACAGGTCTTT
HPRT1	CCAGACAAGTTTGTTGTAGG	TCCAAACTCAACTTGAACTC
COL1A1	TCCCGCCGGTCAAGATGGTC	CGGGCTCTCCAGCAGCACCT
DCN	GGGAGCTTCACTTGGACAACAAC	GGGCAGAAGTCACTTGATCCAAC
LAMA4	GCAGTCCCCAGTCCCGAACG	GGCTGCGCAACCAGCAGAGT
LAMC1	GCCGTGGAGGAGGGCAACTG	CCCCATGGCCAGTGGAACG
COL5A1	CTGCTGCTGCTGCTGTGG	GCGCAAAAGCCTGTTGTCT
COL3A1	GGAGCTGGCTACTTCTCGC	GGGAACATCCTCCTTCAACAG
MIST1	CGGACAAGAAGCTCTCCAAG	CTGGACATGGTCAGGATGGT
LOX	ATGTTGTGCGCTGTGACATT	TTCAGAACACCAGGCACTGA
THBS-1	TGCCTGATGACAAGTTCCAAG	CCAGAGTGGTCTTTCCGCTC
XBP1	CTGGAACAGCAAGTGGTAGA	CTCCTCCAGGCTGGCAGG
